# Anti-obesity and hepatoprotective effects of pyridoxal phosphate in rats with metabolic syndrome by raising anti-oxidant potential in both serum and liver tissue, while also decreasing hepatic nuclear factor expression

**DOI:** 10.22038/ijbms.2025.81836.17702

**Published:** 2025

**Authors:** Sina Mahdavifard, Amir Hasani

**Affiliations:** 1 Department of Clinical Biochemistry, Faculty of Medicine, Ardabil University of Medical Sciences, Ardabil, Iran; 2 Department of Biochemistry and Genetics, Faculty of Medicine, Qazvin University of Medical Sciences, Qazvin, Iran

**Keywords:** Glutathione, Insulin resistance, Metabolic syndrome, NF-kappa B, Pyridoxal phosphate

## Abstract

**Objective(s):**

Insulin resistance is the primary trigger of metabolic syndrome, carbonyl stress, and vitamin B6 deficiency, while the nuclear factor (NF-κB) pathway is a pivotal factor in its development. Hence, we investigated the impact of pyridoxal phosphate (PLP) on liver and kidney functions, carbonyl stress, and inflammatory markers in serum and liver tissue.

**Materials and Methods:**

The study involved four groups of rats, each consisting of eight rats: untreated normal rats (N), rats induced to have metabolic syndrome (MetS), and rats treated with PLP, labeled as N (PLP) and MetS (PLP), respectively. Metabolic syndrome was induced in rats by administering a concentrated sucrose solution for four months. The treated groups received daily PLP at 180 mg/l in their drinking water. Subsequently, the metabolic profile, NF-κB expression, indicators of gly-oxidation, inflammation, and organ function markers were evaluated.

**Results:**

PLP significantly reduced gly-oxidation, carbonyl stress, and inflammatory indicators (in both serum and liver tissue) as well as NF-κB expression, glycation, carbonyl stress, liver fat levels, glycemia, insulin resistance, and body weight (*P*<0.001). The treatment also prevented acute hepatitis.

**Conclusion:**

PLP had beneficial effects in the metabolic syndrome rat model, showing anti-obesity and hepato-renal protective effects. It improved metabolism and organ (liver and kidney) functions by modulating NF-κB expression, glutathione metabolism, carbonyl stress, and oxidative stress.

## Introduction

Metabolic syndrome is a significant issue in clinical practice and public health due to a collection of metabolic factors. It is a predictor of diabetes and cardiovascular complications (1). Obesity serves as a bridge between oxidative stress and inflammation (2). Insulin resistance is the principal component of MetS that leads to its related complications (3). Although many signaling pathways have been introduced for insulin resistance, the pathways involved are still vague. The hepatic nuclear factor (NF-κB) is essential in modulating insulin sensitivity (4). Increasing the hepatic nuclear factor NF-κB pathway signaling and glycation product formations are pivotal participants in insulin resistance, glycemia, and dyslipidemia. 

Insulin resistance causes hyperglycemia, hypertension, dyslipidemia, obesity, and MetS. The hepatic nuclear factor plays a crucial role in liver physiology and diseases. Increasing the nuclear factor NF-κB pathway and the formation of glycation products are pivotal participants in insulin resistance, glycemia, and dyslipidemia. There is a positive relationship between weight and glycation products (5). Glycation products play a crucial role in the progression of insulin dysfunction and metabolic syndrome via NF-κB pathway induction (4). Carbonyl stress, or the accumulation of dicarbonyl compounds such as methylglyoxal (MGO), plays an axial role in glucose intolerance. Glyoxalase-1 (Glo-I) is the cardinal protective system against insulin resistance by lowering dicarbonyl compounds (6). Therefore, inducing Glo-I is an essential strategy in preventing MetS and diabetic vascular complications (7).

Dietary factors are the most crucial consideration in preventing MetS, more so than physical activity (8). Vitamin B6 deficiency is common in metabolic syndrome and may contribute to its development and associated risk factors. Pyridoxal phosphate (PLP) is a vital coenzyme for a wide range of enzymes. Additionally, PLP may help protect cells from oxidative stress due to its strong anti-oxidant properties. PLP deficiency could be linked to hypertension, metabolic syndrome (9), insulin resistance, diabetic complications, atherosclerosis, coronary heart disease, and renal failure (10). Therefore, we researched the impact of pyridoxal phosphate (PLP) on liver and kidney functions, carbonyl stress, and inflammatory markers in serum and liver tissue.

## Materials and Methods

### Materials

All materials were of a high-quality level. 

### Study planning

Male Wistar rats (8 weeks old and 185±10 g weight) were obtained from the Pasteur Institute of Iran in Karaj and were housed under standard conditions. The study was planned on four rat groups, eight rats in each: untreated normal rats (N), metabolic syndrome-induced rats (MetS), and PLP-treated ones, labeled as N (PLP) and MetS (PLP), respectively. Metabolic syndrome in rats was induced by continuously taking a concentrated sucrose solution (40 %) for four months (11). The treated groups received 180 mg/l of PLP in drinking water daily for the same four-month period. The dose of PLP was chosen based on a previous paper (12). Using PLP as a treatment is more effective than vitamin B6 in treating glycation-linked disorders such as MetS and diabetes complications (13). The ethical code of the study was IR.ARUMS.REC.1401. The body weight of rats was measured weekly. Additionally, 24-hour urine samples were collected from them in metabolic cages. After a 16-hour fast, blood samples were collected from the rats’ hearts after anesthesia. Liver tissue was removed, weighed, and homogenized in phosphate-buffered saline. 

*Determination of metabolic parameters*

The metabolic profile, renal dysfunction markers, transaminase activities, and lactate dehydrogenase were determined using Pars Azmoun kits (Tehran, Iran). Sera-free fatty acid (FFA) levels were quantified using high-pressure liquid chromatography (HPLC). 

Fasting insulin was determined with an enzyme-linked immunosorbent assay (ELISA) kit (Mercodia, Uppsala, Sweden). Furthermore, the activities of beta-cells and insulin, represented as %B and %S, respectively, were estimated. 

*Measuring indicators of glycation and Glo-I activity*

Quantifying glycated albumin (g-Alb) was done by measuring the absorbance of reduced nitroblue tetrazolium chloride at 530 nm (14). Glycated LDL (g-LDL) was quantified with a photometric method at 443 nm (15). Methylglyoxal (MGO), as di-nitrophenol hydrazine derivatives, was assessed at 330 nm and determined by HPLC-UV(16). AGEs were measured using a fluorimeter to detect fluorescence intensity (17). The Glo-I activity was measured by detecting the S-D-lactoylglutathione formation at 240 nmol/l (18).

*Measuring indicators of oxidative stress and inflammation in samples*

The serum’s lipid peroxidation marker malondialdehyde (MDA) level was assessed by adding a sample to trichloroacetic acid (TCA). MDA levels were determined by measuring absorbance readings at 535 nm (19). The protein oxidation marker, advanced oxidation protein products (AOPP), was quantified by measuring absorbance at 340 nm in diluted serum (20). The primary LDL oxidation indicator was purified from serum, and its absorbance was measured at 234 nm (21). The terminal LDL oxidation markers were determined with a fluorimeter by measuring the related emission and excitation in nmol/l (22). Measuring glutathione metabolites was done with High-performance liquid chromatography (HPLC) and a UV-detector at 210 nm (23). Paraoxonase-1 (PON-1) activity was quantified by detecting the absorbance of p-nitrophenol at 412 nm (24). Catalase (CAT) activity was determined by measuring absorbance at 240 nm in a phosphate-buffered saline solution of 50 mmol/l containing 10 mmol/L H_2_O_2_ (25). 

An ELISA kits (Immunotech, France) was used for determining interleukin-1β (IL-1β). Myeloperoxidase (MPO) activity was detected by quantifying the absorbance of oxidized guaiacol at 470 nm. 

*The hepatic NF-κB gene expression*

The concentration and purity of RNA were measured following RNA extraction from the tissue using TRIzol reagent. Then, cDNA was produced. Gene expression data were normalized with β-actin (ACTB) as a housekeeping gene. The primers that were used were: 

5´-GGTTACGGGAGATGTGTGAAGATG -3´ (forward)

3´-GGATGATGGCTAAGTGTAGGAC-5´ (reverse) 

ACTB: 5´-GGAGAA GATTTGGCACCACACT-3’ (forward) 3´-CGGTTGGCCTTAGGGTTCAGA-5’ (reverse). 

Finally, the respective gene expression was calculated (26).

*Pathological study*

The 5 μm-thick slices of liver tissue samples were prepared for sectioning and hematoxylin-eosin staining. Additionally, three tissue sections from each rat liver were prepared, and the microscopic examination was conducted three times. 

### Statistical analysis

All data were represented as Mean ± standard deviations and used for data presentation. Using SPSS version 16, a one-way ANOVA test was conducted to compare the variables among the groups. 

## Results

Here, induction of MetS in the rats increased insulin resistance parameters (FBS, HOMA1, and HOMA2). The lowest activities of β-cells and insulin in the untreated MetS group confirm the rise in insulin resistance. Moreover, it elevated the lipid profile, the highest body and liver weight, and their lipid levels. Finally, it increased liver (serum transaminase activity) and kidney dysfunction markers (Cr, PU). Additionally, the highest LDH activity in the group indicates global tissue damage (Table 1). PLP improved organ (liver and kidney) activity. PLP had a beneficial effect on lipid metabolism in treated groups (*P*<0.001*)*. 

**Figure 1:**
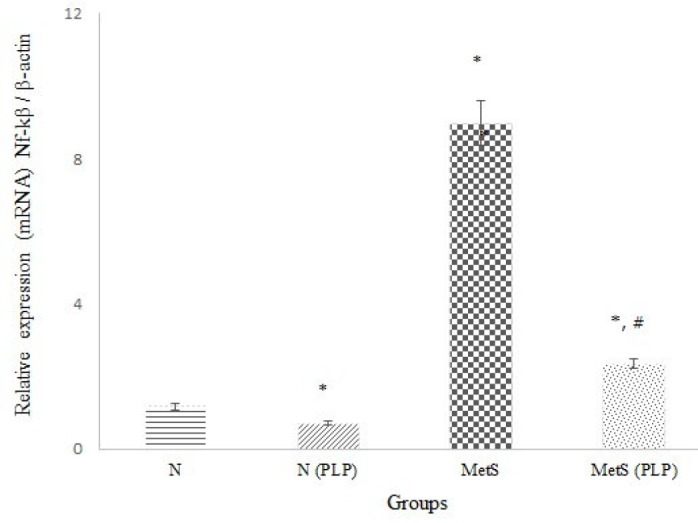
Comparison of hepatic nuclear factor-kβ (NF-κB) relative to β-actin (ACTB) in untreated and pyridoxal phosphate (PLP) treated normal

The N and MetS groups treated with PLP exhibited lower levels of early, intermediate, and end glycation products in the serum. Additionally, they had higher endogenous anti-oxidant levels (glutathione) and anti-oxidant enzyme activities (CAT and PON-1) while showing lower lipid and protein oxidation markers. Moreover, PLP reduced hepatic NF-κB gene expression (Figure 1) and inflammatory indicators in both serum (Table 2) and liver homogenates (Table 3) (*P*<0.001). However, the treatment elevated Glo-I activity solely in the treated MetS group (*P*<0.001).

**Figure 2:**
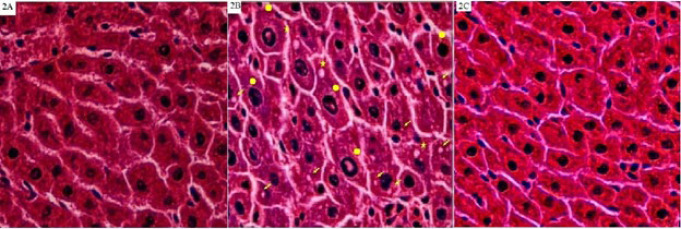
Histopathologic views (stained by H&E & original magnification ×400) of the liver in the untreated and treated with pyridoxal phosphate (PLP), normal (N), and metabolic syndrome (MetS) rats

Metabolic syndrome led to hepatic intracellular inflammation and alteration in liver architecture compared to the N group (Figure 2A). Metabolic syndrome (Figure 2B) led to lipid accumulation (stars), cytoplasmic vacuolization (arrows), and an increasing size of nuclei of hepatocytes (circles). PLP inhibited the modification changes of liver structure due to metabolic syndrome induction (Figure 2C).

## Discussion

Here, increased body weight, insulin resistance, dyslipidemia, and liver fatty changes following a long period of receiving concentrated sucrose solutions (CSS) support the development of metabolic syndrome in rats. Our findings from the MetS rat model align with recent reports (27). Pyridoxal phosphate benefits body weight, insulin function, and lipid metabolism due to its anti-oxidant properties, which help lower glycation and inflammatory indicators. Additionally, the treatment inhibited hepatitis by increasing total and reduced glutathione and decreasing NF-κB pathway expression and its activators. 

Free radicals, persistent inflammation (28), and glycation (29) are critical contributors to the progression of metabolic diseases. Hyperglycemia (30) and vitamin B6 deficiency elevate glycation products (31). Metabolic syndrome causes liver damage with an increase in free radical formation, a decrease in endogenous anti-oxidants, and cell membrane dysfunction. Reactive oxygen species (ROS) can cause liver injury by activating the NF-κB pathway (32). Low levels of GSH are a significant cause of an excessive inflammatory response. Carbonyl stress reduces GSH. Thus, there is an inverse correlation between MGO and GSH (33). A rising GSH/GSSG ratio decreases inflammation by inhibiting the NF-κB pathway (34). Increasing the hepatic GSH/GSSG ratio is an effective plan to protect the liver against metabolic syndrome. Hepatitis in the MetS group due to metabolic syndrome induction was confirmed by observing inflammation (pointed out by arrows) and fat accumulation (Figure 2B, indicated by stars). Additionally, raising the NF-κB expression, transaminases, and LDH activities (Table 2) indicates hepatitis and liver injury. Early to end glycation markers (31) and IL-1β (35) are pivotal contributors to acute hepatitis and insulin resistance by driving hepatic NF-κB signaling. Moreover, oxidized LDL, GSSG, and FFAs activate the NF-κβ pathway (36). Elevating MPO activity results in liver injury by increasing lipid peroxidation (MDA) and protein oxidation indicator (AOPP) (37). An increase in the fat content of the liver by activating the NF-κB pathway participates in hepatitis and insulin resistance (38). Furthermore, a deficiency in vitamin B6 can result in elevated homocysteine levels, which can contribute to metabolic disorders (39) and liver steatosis (40). There is no observation of the fatty changes and inflammation in the liver of MetS (PLP), lower NF-kB expression (Figure 1) and its triggers (Table 2 & 3), the enzyme activities (Table 2), and higher anti-oxidant capacity of the liver (Table 3) and the Glo-I activity satisfies the hepatoprotective and anti-inflammatory properties of PLP and its preventive effect on acute hepatitis. Serum myeloperoxidase (MPO) activity is correlated with obesity, insulin resistance, liver injury, and inflammation. Thus, reducing MPO activity may help prevent or treat obesity, insulin resistance, and inflammation (41). PLP manages body weight and insulin sensitivity by lowering MPO activity in the N and MetS groups. 

A direct correlation exists between levels of different glycation products and body weight (42). Metabolic syndrome induces insulin resistance and dyslipidemia via an increase in several activators of the hepatic NF-kB pathway, including diverse glycation and oxidation products, IL-1β, and FFA (43). In addition, AGEs interfere with glucose uptake and β-cell function (44). Vitamin B6 plays a vital role in the proliferation of β-cells (45) and has an anti-apoptotic effect, decreasing the formation of glycation products and oxidative stress (46). A deficiency in vitamin B6 results in β-cell apoptosis and insulin dysfunction (47). PLP improved glucose metabolism in the MetS group by managing the NF-κB pathway and elevating anti-oxidant indicators in serum and liver. PLP also increased the activity of the anti-glycation enzyme. Pancreatic β-cells are highly susceptible to oxidative stress-induced apoptosis due to producing free radicals and low endogenous anti-oxidant resources (48). An increase in MGO levels leads to GSH reduction (33). PLP, by increasing the GSH/GSSG ratio, may protect against obesity and insulin resistance by reducing oxidative stress and enhancing insulin sensitivity. Recent studies have shown the beneficial effects of vitamin B6 on insulin resistance and liver histomorphology in Apo E−/− mice fed a high-fat diet (49). 

Metabolic syndrome contributes to vascular complications (31). Treatment had anti-atherosclerotic and reno-protective effects due to a beneficial effect on metabolism (Table 2) and a decrease in gly-oxidation markers and inflammation (Table 3). A negative correlation was also observed between PLP supplementation and the cardiovascular index. PLP decreased renal dysfunction markers, including creatinine and urinary protein excretion Cr in the MetS (PLP) group (Table 2). CSS promotes atherosclerosis and nephropathy in this study due to increased free radical formation, inflammation, and LDL gly-oxidation products and decreased Glo-1 activity (Tables 2-4). The higher levels of anti-oxidant profile and lower biomolecule oxidation indicators in MetS (PLP) validate the treatment’s perfect anti-oxidant activity. The literature review shows a direct relation between vitamin B6 levels and anti-oxidant potential (50). A recent report highlighted the beneficial effect of vitamin B6 on the anti-oxidant system by increasing PON-1 activity in diabetic rats. For the first time, a positive impact of PLP on the anti-oxidant potential in MetS rats was observed. PLP reduced LDL modification indicators in both N and MetS rats (Table 3). Here, we reported the effects of PLP on LDL gly-oxidation products or the activities of the anti-oxidant enzymes in metabolic syndrome for the first time. A previous study demonstrated the ameliorating effect of vitamin B6 on hepatic lipid accumulation and dyslipidemia in rats fed a high-fat diet by inhibiting lipid synthesis (51). The impact of PLP on the expression of NF-κB has not been previously documented.

PLP decreased body weight in MetS rats. Formerly, the impact of pyridoxine hydrochloride on reducing body weight in obese and overweight women has been documented (52). 

Hyperglycemia, oxidative stress, glycation, and vitamin B6 deficiency contribute to hypertension. The decline in these risk factors confirms the beneficial effect of PLP, which could potentially improve hypertension. It plays a role in controlling hypertension by regulating cellular calcium transport. A previous study reported that vitamin B6 supplementation attenuated hypertension in spontaneously hypertensive rats (53). One limitation of our research was using only one treatment dose without determining adiponectin levels, glutathione peroxidase, and glutathione peroxidase activities in rats. The lack of blood pressure measurements and the limited sample size were also limitations.

(N) and metabolic syndrome (MetS) rat groups

* Indicates a significant difference from group N (*P*<0.001); # Indicates a significant difference from group MetS (*P*<0.001)

(A) Hepatocytes in the N and N (PLP) groups were polygonal, with round nuclei arranged in plates and interconnected, (B) The liver of the MetS group exhibited fatty changes (stars), cytoplasmic vacuolization (arrows), and hepatocytes with giant nuclei (circles), (C) Evidence of intracellular inflammation was not observed in the livers of rats treated with pyridoxal phosphate (MetS-PLP).

**Table 1:** Comparison of body weight, glucose, lipid metabolism, liver weight index, liver fatty content, and activities of transaminases and lactate dehydrogenase in pyridoxal phosphate-treated normal (N) and metabolic syndrome (MetS) rats

**Parameter**	**Groups (Ten rats in each group)**			
	N	N (PLP)	MetS	MetS (PLP)
**Fasting blood sugar (mmol/l)**	4.96 ± 0.28	4.68 ± 0.28	7.02 ± 0.49^*^	5.87± 0.41 ^*, #^
**Insulin (µU/ml)**	19.32± 1.62	18.10± 1.56	24.96 ± 4.03^ *^	21.04± 2.37^ *,^^#^
**homeostasis model assessment of insulin resistance-1**	4.25± 0.19	3.77± 0.22	7.78 ± 0.73^*^	5.48 ± 0.41^*,^^#^
**homeostasis model assessment of insulin resistance-2**	2.45± 0.06	2.27 ± 0.05	3.40 ± 0.12^ *^	2.77 ± 0.09^*,^^#^
**Percentage of β-cell activity**	180.40 ± 9.80	193.60± 10.14	112.10 ± 3.42^ *^	138.50 ± 6.09^ *,^^#^
**Percentage of insulin sensitivity**	40.80 ± 2.37	44.10 ± 3.39	29.40 ± 1.16^ *^	36.10 ± 1.35^ *,^^#^
**Triglyceride (mmol/l)**	1.49 ± 0.09	1.18 ± 0.08	2.93 ± 0.17^ *^	1.86 ± 0.11^ *,^^#^
**Total cholesterol (mmol/l)**	2.10 ± 0.15	1.77 ± 0.08^ *^	3.56 ± 0.23^ *^	2.49 ± 0.18^ *,^^#^
**HDL (mmol/l)**	1.09 ± 0.08	0.98 ± 0.06	0.81 ± 0.05^ *^	1.14 ± 0.11^ *,^^#^
**LDL (mmol/l)**	0.36 ± 0.11	0.25 ± 0.09	1.41 ± 0.07^ * ^	0.50± 0.11^ *,^^#^
**LDL/HDL**	0.33 ± 0.01	0.25 ± 0.01 ^*^	1.74 ± 0.11^ * ^	0.43 ± 0.02^ *,^^#^
**Free fatty acids (µmol/l)**	582.05 ± 33.27	533.91± 28.42^ *^	802.36 ± 50.97^ * ^	619.03 ± 39.86^ *,^^#^
**Alanine transaminase (U/l)**	26.71± 1.09	23.95± 0.93	91.62± 5.47^*^	38.03± 1.89 ^*,^^#^
**Aspartate transaminase (U/l)**	38.61± 2.44	40.15 ± 2.59	119.74± 7.83^*^	56.07 ± 3.18 ^*,^^#^
**Lactate dehydrogenase (U/l)**	442.53± 22.01	439.34 ± 21.45	762.56± 38.67^*^	550.13 ± 26.94 ^*,^^#^
**Percentage of body weight alternation (%)**	61.03± 3.28	57.16 ± 2.94	140.53± 10.56^*^	81.32± 4.18 ^*,^^#^
**Liver weight index (g/100 g body weight)**	4.25 ± 0.07	4.19± 0.06	5.46 ± 0.15	4.31 ± 0.09^ *^
**Liver total lipids (mg/g liver)**	48.15 ± 3.24	46.86± 3.11	103.75 ± 6.23	60.28 ± 3.30^*,^^# ^
**Creatinine (µmol/l)**	51.38± 3.14	48.20± 2.96	92.83 ± 5.77^*^	69.10± 4.03^ *, #^
**Urine protein excretion (mg/24 hr)**	12.05 ± 0.63	11.74± 0.58	39.23 ± 2.16^*^	24.51 ± 2.44^ *, #^

* Indicates Significant difference with group N (*P*<0.001); # Indicates Significant difference with group MetS (*P*<0.001); PLP: Pyridoxal phosphate; MetS: Metabolic syndrome; LDL: Low density lipoprotein; HDL: High density lipoprotein

**Table 2:** Comparison between serum levels of oxidative stress, inflammatory, and glycation markers in pyridoxal phosphate (PLP) treated and untreated normal (N), metabolic syndrome (MetS) rats

**Parameter**	**Groups (Ten rats in each group)**			
	N	N (PLP)	MetS	MetS (PLP)
**Total glutathione (µmol/l)**	197.01± 12.86	224.28 ± 13.65^*^	136.42± 9.02^*, #^	166.73 ± 10.41^*, #^
**GSH/GSSG **	14.78 ±1.03	16.42± 1.26^*^	8.62± 0.36 ^*^	11.10 ± 1.23 ^*, #^
**CAT (U/mg protein)**	143.03 ±8.22	169.77±11.61^*^	95.66± 6.10 ^*^	118.41 ± 7.04 ^*, #^
**PON-1 (U/mg protein)**	145.52 ±9.64	163.40±12.86^*^	39.30± 2.75 ^*^	118.13 ± 8.01 ^*, #^
**MDA (nmol/l)**	6.18 ±0.45	4.92± 0.57^*^	35.20± 3.64 ^*^	19.34 ± 1.61 ^*, #^
**AOPP (µmol/l)**	15.60 ±1.37	11.28± 1.19^*^	40.93± 3.68 ^*^	27.61 ± 2.44^ *, #^
**IL-1β (Pg./mg protein)**	130.41± 8.40	156.34± 11.96^*^	695.22± 43.84^*^	209.47 ± 13.47^ *, #^
**MPO (U/mg protein)**	0.48 ±0.04	0.28± 0.02^*^	1.87± 0.15 ^*^	0.94 ± 0.07 ^*, #^
**g-Alb (µmol/l)**	102.86 ± 6.38	79.06 ± 3.94^*^	259.07 ± 12.51 *	147.24 ± 9.09 *, #
**g-LDL (µmol/l)**	40.51 ± 2.12	25.08 ± 1.19^*^	98.01 ± 531 ^*^	57.84 ± 2.96 *, #
**MGO (µmol/l)**	13.15 ± 0.60	8.92 ± 0.46 ^*^	45.01 ± 2.10 ^*^	21.74± 1.01 ^*,^^#^
**AGEs (FI, A.U)**	39.46± 1.98	22.07 ± 1.05 *	289.31 ± 16.23 ^*^	78.52 ± 1.62 ^*,^^#^
**ELOP (µmol/l)**	9.83± 0.48	6.09 ± 0.44 *	76.12 ± 3.03 ^*^	36.27 ± 2.03 ^*,^^#^
**FLOP (µmol/l)**	182.03 ± 9.84	147.11 ± 8.62 *	392.01 ± 23.52 ^*^	242.16 ± 13.29 ^*, #^
**Glo-I (U/ml)**	39.26± 1.38	45.01 ± 1.09	20.93± 1.38^*^	31.42 ± 1.96 ^*,^^#^

* Indicates Significant difference with group N (*P*<0.001)

# Indicates Significant difference with group MetS (*P*<0.001)

GSH: Reduced glutathione; GSSG: Oxidized glutathione; CAT: Catalase; PON-I: Paraoxonase-I; MDA: Malondialdehyde; AOPP: Advanced oxidation protein products; IL-1β: Interleukine-1β; MPO: Myeloperoxidase, g-Alb: glycated albumin, g-LDL: glycated LDL, MGO: Methylglyoxal, AGEs: Advanced glycation end products; ELOP: Early LDL oxidation products; FLOP: Final LDL oxidation products, glyoxalase-I: Glo-I

**Table 3:** Effect of pyridoxal phosphate (PLP) on oxidative stress and inflammatory markers in liver (L) and kidney tissue (K) homogenates of rat groups

Parameter		N	N (PLP)	MetS	MetS (PLP)
Total glutathione	L (nmol/mg protein)	519.84 ±26.07	573.20± 29.81^*^	385.39 ± 21.60^ *^	447.65 ± 28.53^*, #^
	K (nmol/mg protein)	348.11 ±21.25	440.54± 30.21^*^	166.83 ± 9.01^ *^	352.70.25 ± 21.72^*, #^
GSH/GSSG	L	15.96± 1.08	17.84± 1.57^*^	7.05± 0.12^*^	9.63 ± 0.46^ *, #^
	K	8.76± 0.58	9.58± 0.72^*^	4.10± 0.31^*^	7.03 ± 0.53^ *, #^
CAT	L (U/mg protein)	8.16± 0.45	9.01± 0.61^*^	4.67± 0.37^*^	6.91 ± 0.43^ *, #^
	K (U/mg protein)	17.10± 0.92	25.89± 1.83^*^	4.50± 0.39^*^	12.04 ± 0.88^ *, #^
PON	L (U/mg protein)	8.94± 0.59	10.83± 0.74^*^	4.79± 0.32^*^	6.88 ± 0.47^ *, #^
MDA	L (nmol/g tissue)	6.18 ±0.45	4.92± 0.57^*^	35.20± 3.64^ *^	19.34 ± 1.61^ *, #^
	K (nmol/g tissue)	5.06± 0.39	3.99± 0.25^*^	19.28± 1.20^*^	10.13 ± 0.67^ *, #^
AOPP	L (nmol/g tissue)	10.18± 0.62	6.79± 0.03^*^	51.45± 3.28^*^	29.21 ± 3.20^ *, #^
	K (nmol/g tissue)	8.26± 0.57	5.01± 0.34^*^	27.53± 1.94^*^	15.62 ± 0.71^ *, #^
MPO	L (U/mg protein)	0.57± 0.05	0.36± 0.03^*^	2.35± 0.21^*^	1.12 ± 0.09^ *, #^
	K (U/mg protein)	0.41± 0.03	0.27± 0.02^*^	2.01± 0.13^*^	0.94 ± 0.05^ #^

* Indicates significant difference with group N (*P*<0.001)

# Indicates significant difference with group MetS (*P*<0.001)

GSH, reduced glutathione; GSSG, oxidized glutathione; CAT, catalase; PON, paraoxonase; MDA, malondialdehyde; AOPP, advanced oxidation protein products; MPO, myeloperoxidase

## Conclusion

PLP had a positive impact on a MetS rat model, showing anti-obesity, anti-atherosclerotic, and hepato-reno-protective effects. It improved metabolism and organ function. Glutathione and anti-oxidant enzymes likely play a key role in protecting against metabolic syndrome. 
